# District of Columbia

**Published:** 1897-08

**Authors:** 


*District of Columbia.* A bill has been introduced into Congress providing for the examination of animals supplying milk to the citizens of the District of Columbia, and to insure the supply being from animals free from tuberculosis; all animals to be examined and tested by veterinarians.

